# Responding to the COVID-19 emergency: student and academic staff perceptions of academic integrity in the transition to online exams at three Australian universities

**DOI:** 10.1007/s40979-021-00075-9

**Published:** 2021-03-26

**Authors:** Alison Reedy, Darius Pfitzner, Laura Rook, Leonie Ellis

**Affiliations:** 1 Charles Darwin University, Darwin, Northern Territory Australia; 2 University of Wollongong, Wollongong, New South Wales Australia; 3 University of Tasmania, Tasmania, Australia

**Keywords:** Academic integrity, Cheating, Online exams, COVID-19, Pandemic

## Abstract

This paper explores the perceptions of academic staff and students to student cheating behaviours in online exams and other online assessment formats. The research took place at three Australian universities in July and August 2020 during the emergency transition to online learning and assessment in response to the COVID-19 pandemic. The study sought to inform decision making about the future of online exams at the participating universities. Quantitative and qualitative data were collected using online surveys. The findings of the study led to seven key observations, most notably the need to redefine the characteristics of academic misconduct to account for changes wrought to examinations in a digital world. The study concludes with lessons learned in relation to enhancing academic integrity in digital examinations and assessments.

## Introduction

In this paper we explore academic staff and students’ perceptions to cheating during the COVID-19 pandemic. The fast-paced transition at scale to online exams during the extraordinary circumstances of 2020 provided a unique opportunity to compare different approaches taken by three Australian universities to moving traditional invigilated exams into online exams or alternative assessment formats and build a picture of staff and student experiences. The authors saw an opportunity to document and research the use of online examinations during times of emergency, and in so doing to establish an evidence-base to guide iterative improvement in their design and deployment during the pandemic and beyond. While two of the three universities had trialled online proctoring prior to the COVID-19 pandemic, the widespread shift to online exams and alternative assessment approaches across the sector, and the variety of approaches taken by different universities, provided a unique opportunity for research. For the purposes of this paper we use Allan’s (2020) understanding of online exams to be “high-stakes summative assessment events, mediated by digital technologies, often taking place in a defined place or time and under secure conditions (e.g. invigilation, restrictions on access to course materials, notes or communication)” (Allan 2020 p 1).

The crisis hastened the movement towards online assessment, including the use of online proctoring, that was already happening in the higher education sector in Australia (Cramp et al. 2019; Day and Lawrence 2020; Hillier 2014; Ladyshewsky 2015), with technological solutions used to migrate previously hand-written exams into online environments (Allan 2020). The benefits of migrating examinations online included spatial and temporal flexibility for students as well as cost benefits and a reduced administrative burden on institutions. Those benefits of online examination that were evident prior to the pandemic (Hillier 2014; James 2016) were highlighted by movement restrictions to control the spread of the pandemic that occurred in Australia and around the world. Some of the challenges that were experienced in the rapid transition to online assessment alternatives in response to the pandemic have previously been identified in different contexts, such as privacy concerns and technical issues with online proctoring (James 2016; Sullivan 2016), issues of digital equity (James 2016), and concerns about academic integrity in online environments (Boitshwarelo et al. 2017; Selwyn 2008a, 2008b; Sullivan 2016).

While the scale of the movement to online exams during the pandemic was unprecedented and unexpected, it provided a context where institutions and individuals, both students and staff resistant to online assessment may have had the opportunity, or “felt compelled to embrace the digital academic experience” (Mishra et al. 2020 p 2), leading to improvement of their “technological educational skills” (Elzainy et al. 2020). That is, the pandemic stimulated the unfreezing of existing pedagogical practices according to Lewin’s (1958) three phase process for change management: “unfreezing changing refreezing” (Mishra et al. 2020). The unfreezing and change in pedagogical practices that occurred through the use of technology in response to the pandemic cannot be reversed. It is likely that the individual and institutional learnings generated by the crisis continue to inform post-pandemic decisions about online assessment, especially given that online assessment was already “becoming more common practice” (James 2016 p 1) in the years prior to the pandemic.

This paper begins by reviewing literature relating to online examinations and academic integrity before introducing the context of the study and the research design. The findings lead to seven key observations which are raised in the discussion, one of which points to the limitations of traditional conceptualisations of academic misconduct in relation to digital examinations. We conclude with lessons learned for enhancing academic integrity in digital examinations and assessments.

## Literature review

An impact of the COVID-19 pandemic in 2020 on higher education was the rapid transition from face-to-face invigilated exams to online exams at many universities around the world (Grajek 2020; UNESCO 2020). While there was also some shift to alternative assessment forms (Bearman et al. 2020; Grajek 2020), in many disciplines and nations, high-stakes summative testing in the form of examinations continues to be the accepted form of assessment (CPA Australia n.d.; Raaheim et al. 2019). Invigilated examinations are used because they are “useful logistically and for assuring the public that plagiarism is under control” (Biggs 2001, p. 234) although there is longstanding concern that “invigilated examinations are hard to justify educationally” (Biggs 2001, p. 234).

Indeed, there is ample research indicating that approaches such as invigilation are needed as breaches of academic integrity are a significant problem in Australian higher education (Bretag et al. 2014) with self-reported incidences of plagiarism reportedly as high as 81% (Marsden et al. 2005). The issue of academic misconduct may be the “*most commonly reported challenge in online assessment*” (Hollister, Berenson 2009 p 272), although studies have also found that cheating may be reduced in online environments as some students are less inclined to cheat in digital environments because of a greater fear of detection (Selwyn 2008a).

There is concern that using technological approaches to replicate high stakes examinations in proctored online environments does not solve the underlying “*social problem of cheating*” (Stockwell 2020, last sentence) and that as new technological solutions are put in place “*students find new ways to cheat*” (Joel 2020 7 paragraphs from end). Mitigation strategies aimed to counter academic misconduct in online assessment include low tech interventions such as proctoring or the use of time-stamps on assignment submissions and high tech interventions such as “*engaging biometrics, such as fingerprint scans, optic–retinal tests, facial recognition, and keystroke pattern analysis*” (Sullivan 2016 p 196). Most success in countering cheating is realised when technological solutions are used along with approaches to enhance integrity and “*ethical consciousness*” (Sullivan 2016 p 195) by merging the concept of academic integrity with personal integrity and “*ideas of social responsibility*” (Abdalqhadr 2020 p 93). The need for education around academic integrity extends to staff (Curtis et al. 2021) as much as it does to students in order to develop consistent understandings of academic integrity across all stakeholders within an institution.

In Australia, the transition to online examinations due to COVID-19 was supported by the higher education regulatory body, the Tertiary Education Quality and Standards Agency (TEQSA), if changes in modality were “*in the best interests of students and the quality of learning*” (TEQSA 2020 paragraph 3). Australian universities had flexibility to determine their individual approaches to online teaching and assessment, including what alternatives they would use to replace high stakes invigilated examinations. Research has found “*no statistically significant difference in the students’ academic achievement in online and traditional exams*” (Ilgaz and Afacan Adanır 2020 p 1255), however, the decision to move examinations online requires judgement about the relative merits of different assessment and examination types in securing the integrity of the assessment or exam environment. Given the speed needed to respond to the changing educational environment because of the pandemic, many Australian and New Zealand universities substituted traditional face-to-face invigilated examinations with their digital equivalent, proctored online exams (Sankey 2020). Over three quarters of universities were predicted to use online proctoring in Semester 12,020 examinations (Grajek 2020), although a subsequent survey of public universities in Australia and New Zealand conducted after that examination period reported that 51% used online proctoring solutions and 49% moved to alternate modes of examination or assessment (Sankey 2020). Some universities that did use online proctoring chose to minimise its use to where it was “*completely necessary”* (Sankey 2020 p. 2), although the use of proctoring does not negatively impact on student performance (Rios and Liu 2017).

The significant use of online proctoring services by Australian and New Zealand universities during the pandemic is despite ethical, equity and technical issues with online proctoring (Allan 2020; Cramp et al. 2019; Grajek 2020; James 2016). An additional concern is that the effectiveness of online proctoring in curbing cheating behaviours is unclear. While there are studies that suggest that cheating increases when online exams are not proctored (Harmon and Lambrinos 2008; Reich et al. 2018), other studies indicate that cheating behaviours are not linked to surveillance but to the nature of the exam itself, with cheating less likely to take place when authentic forms of assessment are used (Bearman et al. 2020; Harper, Bretag, Rundle 2020; Harrison 2020). This is despite authenticity alone not being a panacea to academic misconduct (Ellis et al. 2020). The effectiveness of online proctoring is further put in doubt by student posts on how to cheat in proctored online exams (Blumenfeldwitz 2020).

Securing integrity in digital examinations is conceptually different to traditional approaches, with research indicating that categories of academic misconduct and their definitions need to be reconsidered for the digital age (Evering and Moorman 2012; Sidi et al. 2019). The International Network for Quality Assurance Agencies in Higher Education (INQAAHE) identifies six behaviours that are commonly regarded as constituting academic misconduct. They define one of these, impersonation, as: “*falsely presenting oneself, or engaging someone else to present as oneself, in an in-person examination*” (INQAAHE 2020, p. 5, our emphasis). This definition clearly does not consider the context of digital examinations and evidences the need to rework definitions of academic misconduct (Sidi et al. 2019).

The nature and incidence of academic misconduct is changing with widespread use of digital technology. An increase in plagiarism is “*directly associated with technology, due to the easy access to information and the ease of copying and pasting, which makes data more easily accessible and transferrable than it is in the analog medium*” (Sidi et al. 2019, p. 3309). In contrast, other forms of academic misconduct are higher in face-to-face as compared to digital environments (Sidi et al. 2019).

The digital age has also created a generational rift in perceptions of academic integrity, with young people perceiving “*knowledge ownership, acquisition, and distribution in radically different terms than in previous generations*” (Evering and Moorman 2012, p.35). This has resulted in vastly different perceptions between staff and students of the seriousness and appropriateness of penalties for different forms of academic misconduct (Busch, Bilgin 2014) which is exacerbated by confusion stemming from lack of clarity or ambiguity in academic integrity policy and definitions of cheating behaviours (Merkel 2021; Owunwanne et al. 2010; Ray 2020; Sutton and Taylor 2011). Additionally, educators and students are increasingly confused about what constitutes academic misconduct in the digital age, which Hamblin (2017) refers to as the ‘blurred lines’ of academic integrity when digital technologies are added to the mix. This confusion is further exacerbated by different disciplinary traditions and approaches to integrity and the distinct cultures of academic integrity that they generate (Sutherland-Smith 2013).

## Context of the study

Three Australian universities participated in this research and are referred to as universities ‘A’ ‘B’ and ‘C’ in this paper. Universities A and C are located in small capital cities and university B is in a large regional centre. Prior to the pandemic, university A was an established provider of online education while the other two universities used face-to-face approaches to teaching and learning.

In response to the COVID-19 pandemic all three universities moved from face-to-face invigilated examinations to online exams and alternative assessment approaches in Semester 1, 2020. These were deployed through each university’s Learning Management System (LMS). At university A, online exams as well as other forms of online assessment were adopted but online proctoring and browser lockdown software were prohibited. At university B, non-exam formats of assessment were mandated with exams only permitted by exception and with permission. At university C, staff were told to find alternative forms of assessment to replace exams, and online proctored exams were supported only if required by the accrediting body. At all three universities the changes brought on by the pandemic resulted in a decrease in examinations. For example, at University C, 285 exams were conducted in Semester 1, 2020 as compared to 432 in Semester 1, 2019.

Consideration of academic integrity played out differently at the three universities during the pandemic. This reflects the diversity of approaches that universities and faculties in the sector take to address academic integrity, spanning from teaching and learning focused approaches (Bertram Gallant 20,017) to approaches that feature “moralistic and regulatory discourses” (Hu and Sun 2017 p 56).

At university A different approaches to countering academic misconduct were taken by different disciplines. For example, in one faculty, students had to read and accept a declaration of academic integrity before their exam was released to them online. Additionally, completed exam papers were submitted through text-matching software. Some exams at all three universities were delivered through a test tool in their LMS, mainly using multiple-choice questions, with deployment setting such as randomisation used to maximise individualisation and minimise opportunities for collusion. While this study compared the broad approaches to online examination format and deployment at the three universities, more detailed examination of individual examinations is needed to deepen our understanding of which specific approaches to online examinations were successful and for whom.

## Research design

This research was conducted as an exploratory study in the context of the pandemic. The study was developed rapidly in order to collect data about the first large-scale rollout of online examinations at the three participating universities. The aim of the study was to understand what worked in the scale up to online examinations in response to the COVID-19 pandemic and what could be improved for subsequent deployment of online examinations and alternative assessments. The study sought to understand:

- What are the experiences and perceptions of students and academic staff of final examinations conducted in Semester 1, 2020 as a response to the COVID-19 pandemic?
- How do these experiences vary across the three universities?
- How may these experiences inform the development of online examinations post-pandemic?

Within the overall experience of online examinations, the study asked students and staff about their perceptions of the ease of cheating in online exams and other assessment formats that were used as alternatives to invigilated online exams in Semester 1, 2020. Ethics approval for the study was granted at each of the participating universities.

## Method

Data were collected from academic staff who were directly involved in teaching and assessment (i.e. excluding academic management) and students. Online surveys were developed and deployed using Qualtrics software. All questions were voluntary/opt-in and exit anytime.

The student survey (Appendix 1) contained thirty-one questions about student experiences in the transition from traditional invigilated exams to online examinations (proctored and non-proctored) or alternative forms of assessment. The survey contained 18 default questions for all participants plus 13 conditional questions that were asked depending on prior answers. The online staff survey (Appendix 2) also contained 31 questions, with 13 default questions and 18 conditional questions. Specific questions were asked about the types of alternate assessments used, assessment deployment conditions, training, information supplied leading up to and during the assessment, and perceptions of cheating relative to assessment type. The surveys were anonymous, voluntary, and contained both quantitative and qualitative (open response) question types.

### Analysis

Before conducting any analysis, all data were cleaned, resulting in the elimination of 318 student responses and 24 staff responses. This was achieved by deleting all response sets that only contained responses to Questions 1 to 9 (primarily demographic in nature) or junk, such as random characters, in the open style questions.

Given the investigative nature of this research, the quantitative results are reported based on summary and distributional statistics. The quantitative data from the surveys was downloaded into a spreadsheet for post processing, analysis and the creation of data representations (e.g. tables, plots and graphs).

Importantly, qualitative data drawn from the survey was used to unpack the stories behind the numbers. The analysis of the qualitative data was “*a dynamic, intuitive and creative process of inductive reasoning, thinking and theorizing*” (Basit 2003, 143). Qualitative data from the surveys was download into a spreadsheet and manually coded using deductive reasoning based on the questions asked, and inductively as unanticipated themes emerged. The alignment of the data to survey questions made the coding relatively straightforward. While questions that asked specifically about perspectives on cheating behaviours were the main source of data, responses to other qualitative survey questions were also reviewed.

### Participants

Academic staff from the participating universities were contacted by email and invited to participate in this study if they had transitioned from a traditional invigilated examination planned for Semester 1, 2020 into an online format or alternative assessment type. A total of 73 academic staff accepted this invitation with 49 of these responses retained after data cleaning, which eliminated incomplete responses.

Students at the three universities whose assessment had changed in Semester 1, 2020 from a planned invigilated exam to an online format of exam or assessment were also invited by email to participate in the study. Two approaches were used by the different universities, these were: (i) email lists of all exam participants were supplied by student central /examinations office, and (ii) automated student communication systems were used to advertise the survey. 2239 students accepted the email invitation, of which 1921 usable responses were analysed.

Of the students who participated in the study 61% (*n* = 1175) were female, 38% (*n* = 731) were male and 1% (n = 11) identified as non-binary. The usual mode of study of participants (prior to the pandemic) was 44% (*n* = 844) internal (campus-based) and 56% (*n* = 1077) external (online). 66% (*n* = 1266) of participants were domestic students while 34% (*n* = 655) had international status. The majority of participants in the survey, 74% (*n* = 1414) were undergraduates, with a smaller proportion of postgraduate students, 26% (507). The age distribution of participants shows the highest number of participants in the 18 to 24 age group, with participation falling with age.

### Limitations

Three categories were used in the staff and student surveys to identify different types of online exams and assessment types. These were developed at the same time the participating universities were deciding what approaches to take to replace traditional invigilated examinations. With hindsight, the assessment types and the terms used to describe them could have been further refined to provide more nuanced data across exam and alternative assessment types. The difficulty in refining and classifying types of online exams is the diversity in online assessment formats that range “from online essay submission to fully automated, computer-marked online examinations” (James 2016 p 1).

A second limitation was the collection of demographic information by course of enrolment rather than discipline of the exam. This limited the ability of applying inferential statistics on the basis of discipline.

A third limitation was the limited number (*n = 49*) of valid staff responses.

## Findings

In this section we present the findings from the student and the staff surveys, starting with an overview of the quantitative data and then moving on to the qualitative data.

**Fig. 1:**
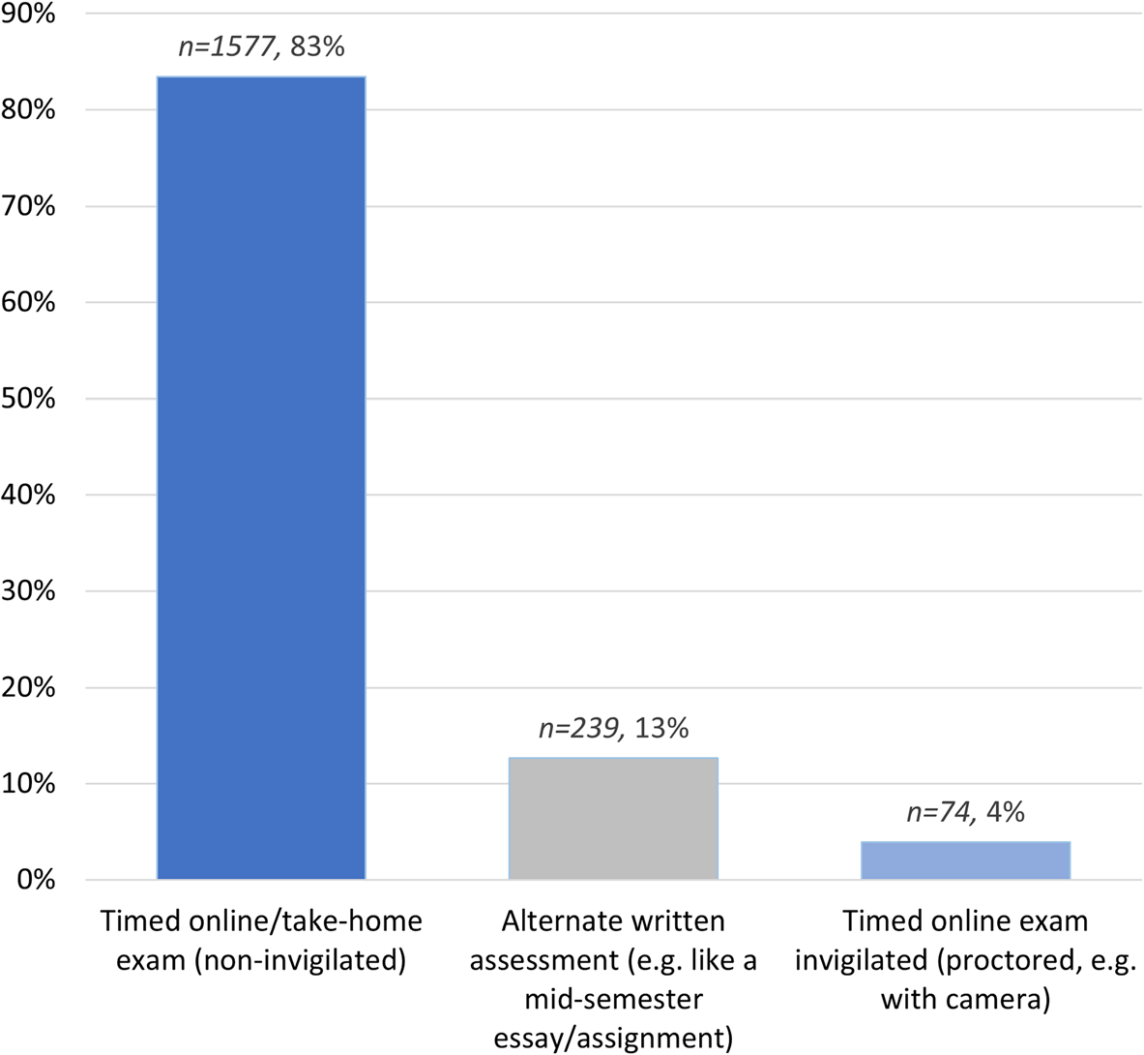
Incidence of online assessments or exam format that replaced traditional invigilated exams

Most students (*n = 1577*, 83%) had a traditional invigilated exam replaced by a non-invigilated timed online exam including time-limited take-home exams (see Fig. 1). The term ‘take-home exam’ is conflated with non-invigilated timed online exams to mirror the nomenclature used at one of the universities. A small proportion of students (*n = 239*, 13%) had traditional invigilated exams replaced by alternate written assessments, such as an essay or assignment. Very few students (*n = 74*, 4%) had an online timed invigilated/proctored exam.

**Fig. 2:**
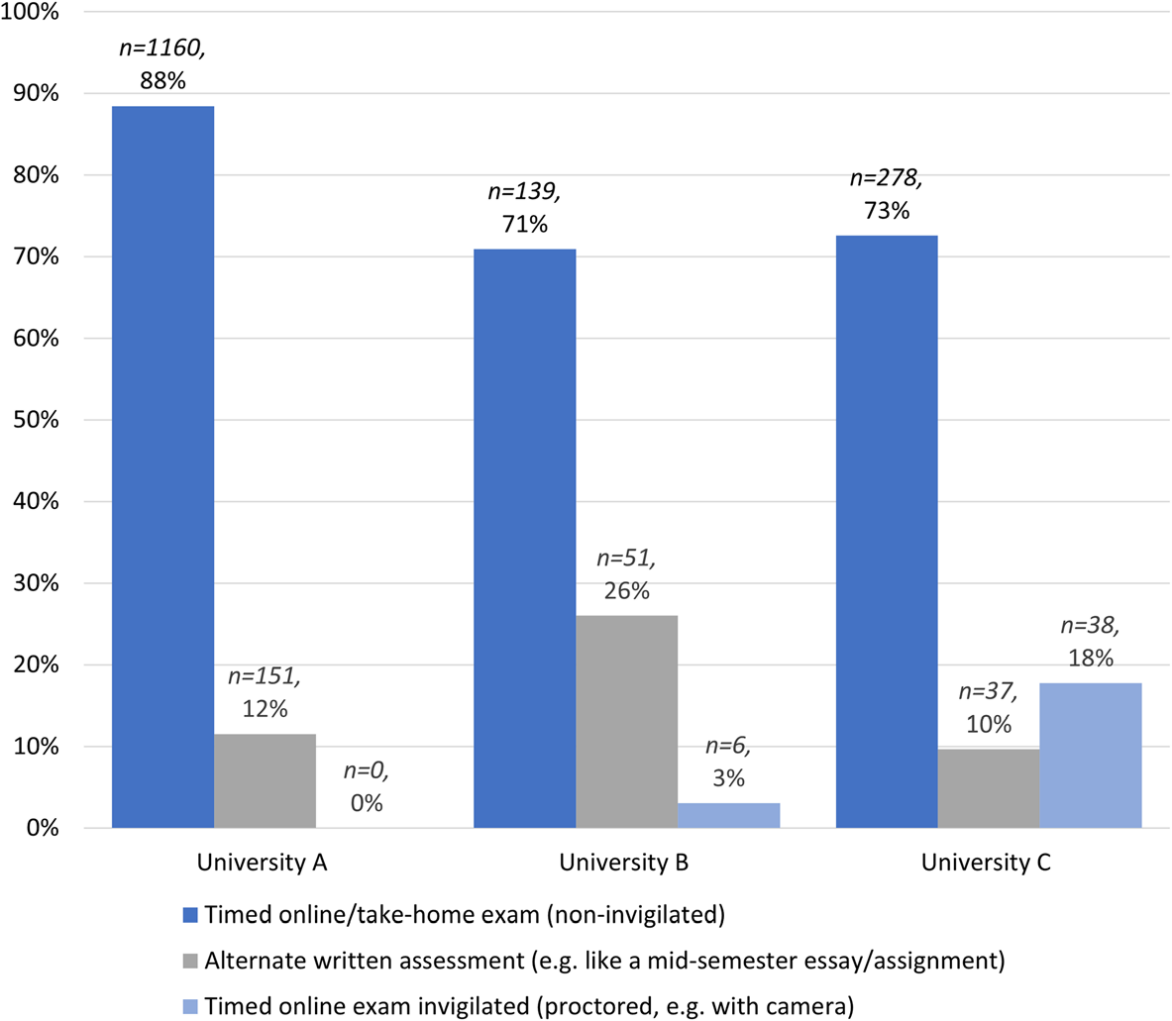
Distribution of online exam type or alternate assessment by university

Non-invigilated timed online exams were the most common technique used (see Fig. 2). Only one of the three universities advised that they used online proctored exams, although students at two of the universities indicted that they had sat proctored exams.

While timed online non-invigilated exams were used by all disciplines and universities, some examinations at universities B and C were proctored. Proctored examinations were used in a wide variety of disciplines (Table 1) which was inconsistent with the position of all universities that online proctored exams were not to be used, or only used if required by an accrediting body.

**Table 1:**
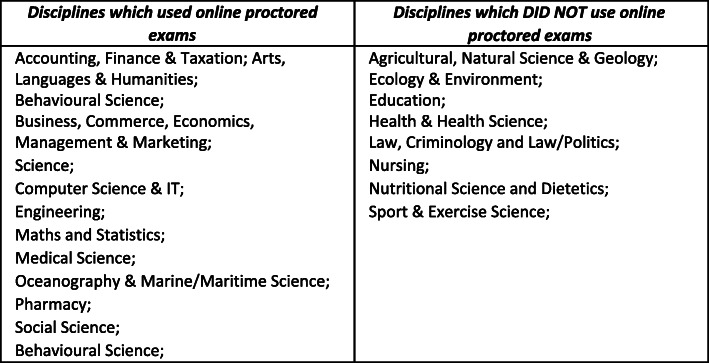
The use of online proctored exams by discipline at University C

**Fig. 3:**
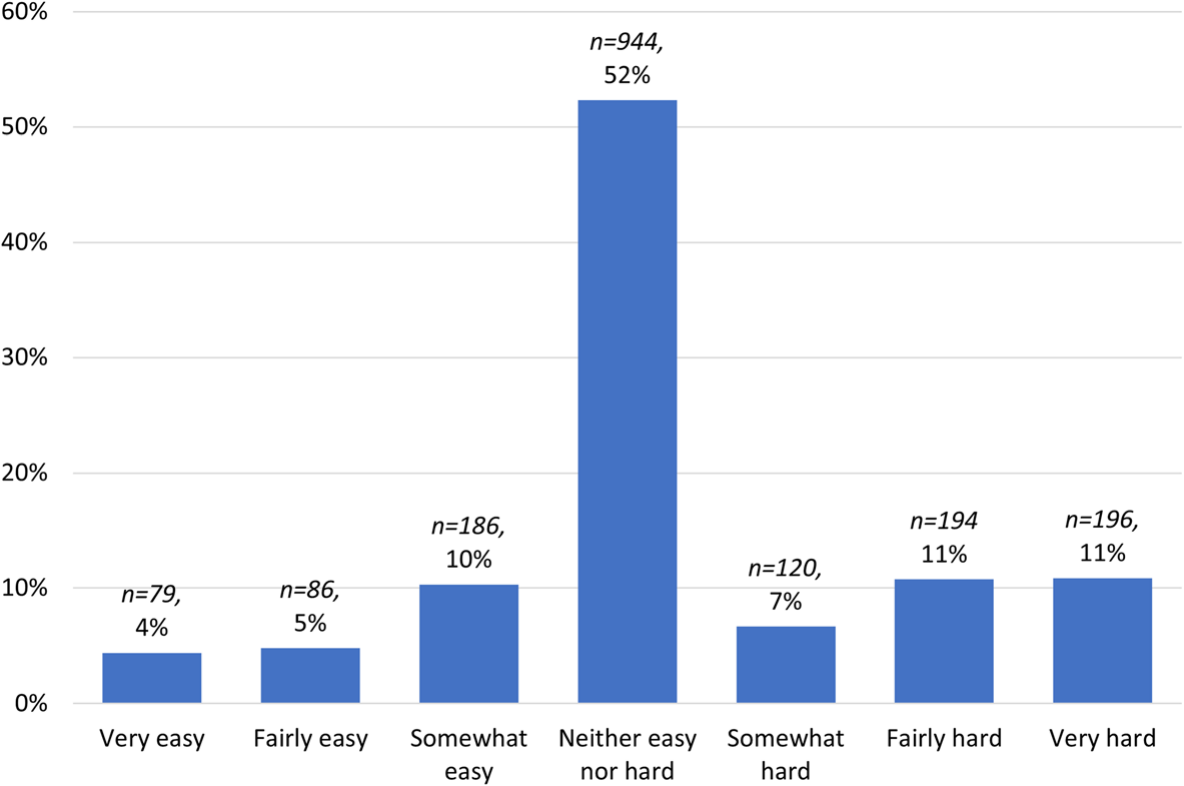
Student responses to the statement: “The online exam or alternate assessment made cheating … ”

Students and staff were asked if they perceived that cheating was harder or easier in the online exam or alternate assessment format than in a traditional invigilated exam. Students’ responses to that question (*n = 1827*) were gauged on a 7-point Likert scale (see Fig. 3). More than half of students (*n = 944,* 52.27%) perceived that there was no difference in the ease of cheating between a traditional invigilated exam and an online exam or alternate online assessment, and more students perceived it to be harder (*n = 510,* 28.30%) rather than easier (*n = 351,* 19.43%) to cheat in an online environment than in a traditional face to face invigilated exam.

As discussed by Holden et al. (2020), the literature is inconclusive about whether cheating is more prevalent in online or face-to-face exams. The findings from our study align with those from a study undertaken by Sidi et al. (2019) that found that “digital academic dishonesty was less pervasive and deemed more legitimate compared to analog dishonesty” (p 3300). However, those findings contrast with the more pervasive view that cheating is easier in online examinations than in face-to-face exams (Chirumamilla et al. 2020). The variability of findings between studies points to the importance of context and the nature of the exam or assessment type on student perceptions of the ease of cheating. Additionally, perceptions of ease of cheating in online environments changes by the nature of the assessment or exam as well as the type academic misconduct. For example, plagiarism is easier in online assessments (Sidi et al. 2019), whereas using “forbidden aids” (Chirumamilla et al. 2020 p 940) is a form of academic misconduct of more concern in online exams.

**Fig. 4:**
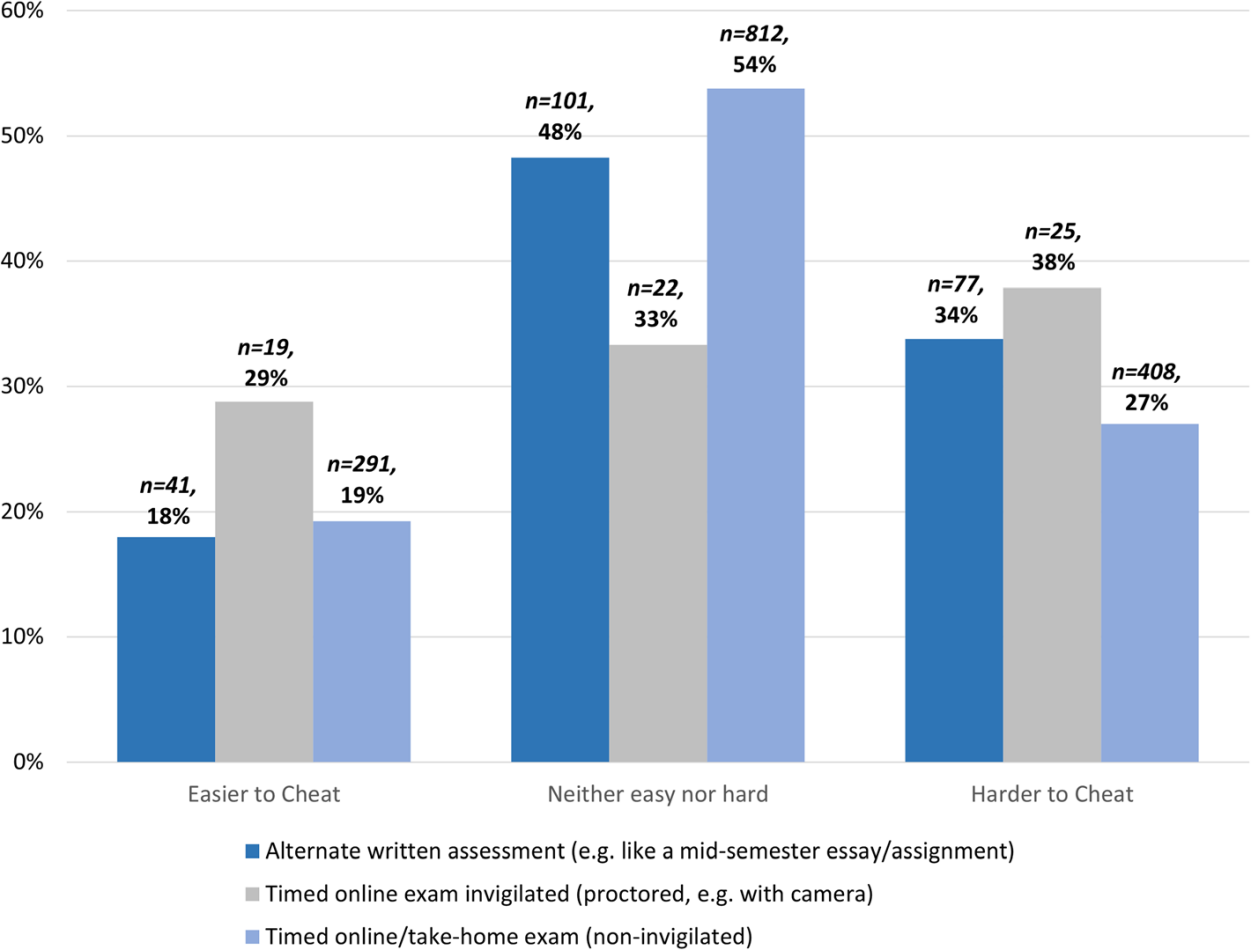
Perceptions of cheating by exam or assessment format

In terms of exam format, significantly more students thought it was harder rather than easier to cheat in online exams and alternative assessments relative to traditional invigilated exams across all online examination and assessment formats (see Fig. 4).

**Fig. 5:**
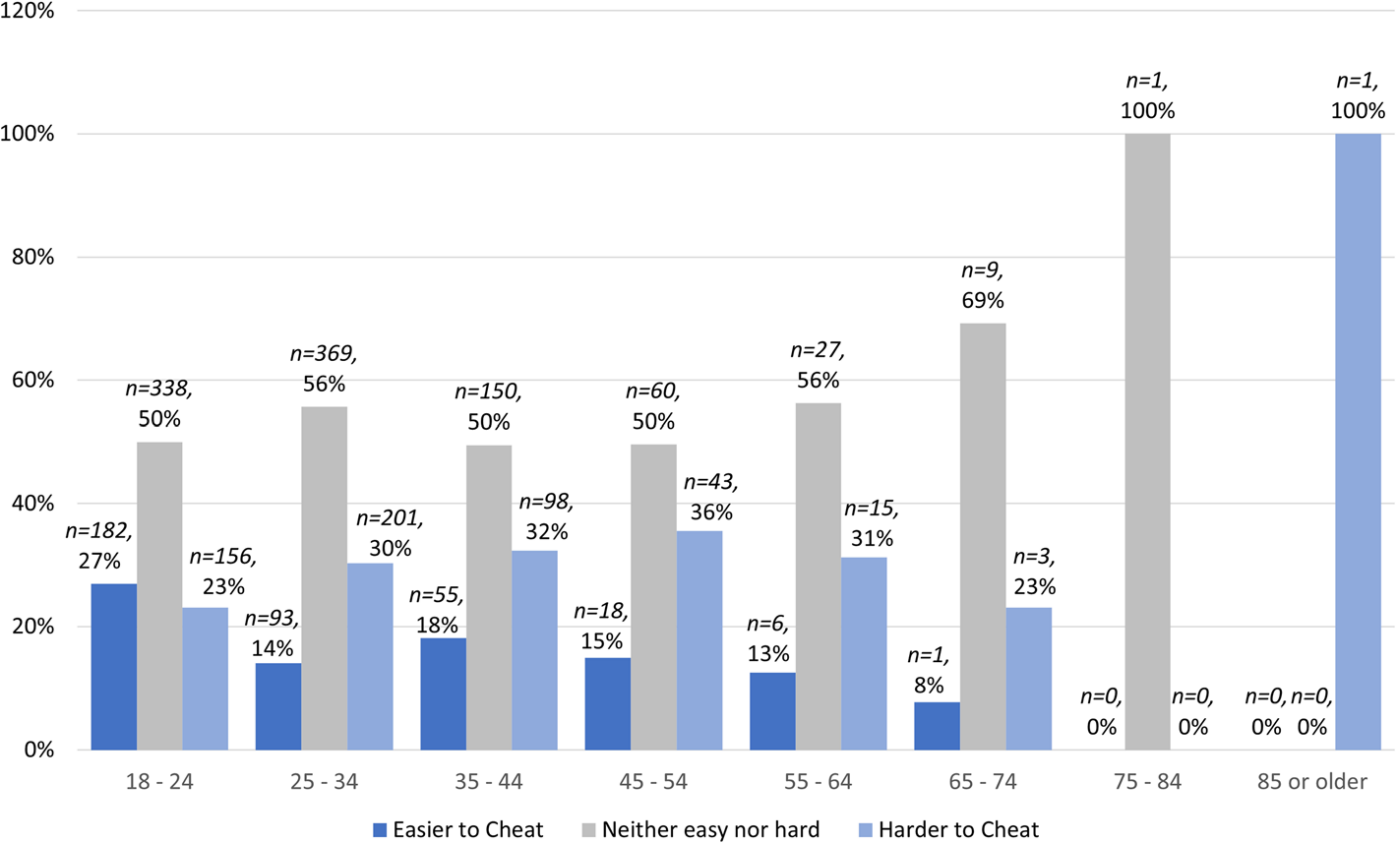
Perceptions of cheating by age

The data were also analysed to see if student perceptions of academic integrity were impacted by age, gender, level of course (undergraduate or postgraduate), or nationality (domestic or international student). Age was a significant factor in student perceptions of cheating (see Fig. 5), a factor in common with other studies (Tremayne and Curtis 2020). In contrast to other studies (Beasley 2016; Tremayne and Curtis 2020), this study found no significant difference across the other demographic categories in terms of perceptions of cheating.

Younger students, in the age range 18–24 years (*n = 676*), perceived cheating to be easier in online exams and alternative assessments than any other age group. However, in this age group there was also a relatively even distribution (*n = 26,* 4% difference) between those who perceived it was easier to cheat (*n = 182*) in online formats as compared to traditional invigilated exams, and those who thought it was harder (*n = 156*). This difference in perception and the tendency to consider cheating to be harder generally increased with age, with students aged 65 and older (*n = 17*) being the least likely to consider cheating to be easier in online formats. In all age groups apart from the youngest students, there was a perception that it was more difficult to cheat in online exams or alternative assessment formats than in traditional invigilated exams, while 50% or more of all age groups less than 65 (*n = 1811*) considering that exam modality made no difference to the ease of cheating.

In comparison, staff perceived that it was easier for students to cheat in online exams and assessment than in traditional invigilated exams and expressed concern about an increase in student cheating behaviours with the move away from traditional invigilated examinations to digital exams and assessments. This perspective is mirrored in other studies of staff perspectives of cheating, which suggest that up to 93% of staff surveyed believe cheating is easier for students in online environments (Lederman 2020; Wiley 2020).

**Fig. 6:**
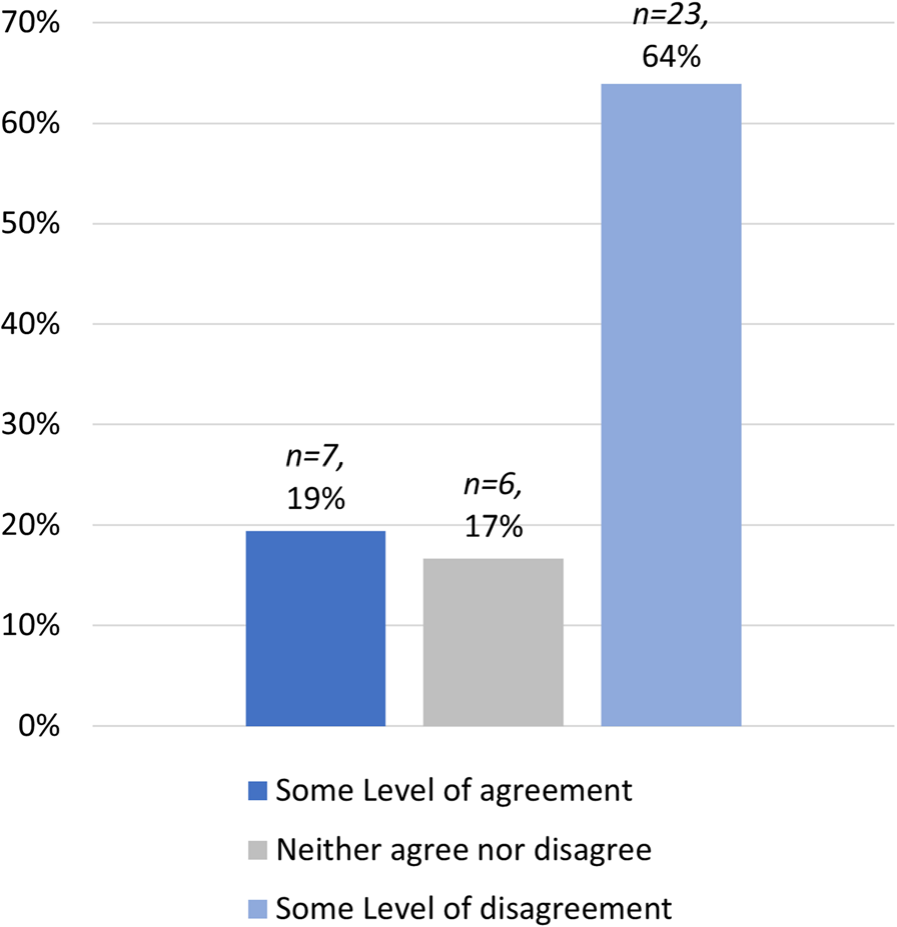
Perceptions of cheating: Staff levels of agreement with the statement that cheating is minimised in online exams and assessment as compared to traditional invigilated exams

In our study 64% (*n = 23*) of staff disagreed with the statement that student cheating is minimised by the online format of the exam or assessment that they used in Semester 12,020 (see Fig. 6). This perception by staff that exam format does not impact on student cheating behaviours contrasts with evidence that points to the importance of exam format in reducing academic misconduct (Bearman et al. 2020; Harper, Bretag, Rundle 2020; Harrison 2020). This suggests that staff development is needed at the participating universities to provide education on exam design that minimise the incidence of academic misconduct (Brimble 2015; Curtis et al. 2021).

### The nature of digital cheating

The study did not define the term ‘cheating’ and left it open to respondents’ interpretations. The main ‘cheating’ behaviours identified by students were accessing resources, collusion, and impersonation. Staff identified similar concerns plus that of contract cheating.

#### Access to resources

A common concern for students and staff was cheating that involved access to resources during the exam that were not permitted, which is a concern echoed in the literature (Chirumamilla et al. 2020; Dendir and Maxwell 2020). For some students, the use of any resources other than retained knowledge, such as “*text books and computers,”* was linked to a perception of cheating. This is evident in the comment: “*We are allowed to do an open book exam, which means somewhat cheating I think*.” Academic staff were concerned that when doing exams online from home, students could “*use whatever resources they like (possibly including another person)*” and there was nothing to stop them from doing *so, “i.e., they all seem to be open book exams*.”

Students were confused about what resources were permitted in online exams, and they individually interpreted what resources they could access and what constituted cheating. This confusion extended to what resources could be used during ‘closed book’ exams as well as ‘open book’ exams and uncertainty about what resources were “forbidden aids” (Chirumamilla et al. 2020 p 940).

“*My exam said it was closed book. I followed this rule despite not being sure of some answers. Being able to check my book or discuss the paper with another student would have been quite easy as it was not monitored like the mid semester exam using locked down browser. I think clearer guidelines as to what was expected should have been provided, as in, were we allowed to look at books etc. obviously not talking to other students*”

The lack of clarity in examination instructions about what resources could be used contributed to confusion about what constituted cheating. This exacerbates pre-pandemic confusion about the specific practices that are breaches of academic integrity (Starovoytova and Arimi 2017). In addition, the use of widely understood terms in the context of traditional face-to-face examinations such as ‘open book’ or ‘closed book’ were unclear in the context of the online environment, where students had access to digital resources and search engines. The dissonance between terms used in digital and face-to-face learning contexts highlights the need for changes in assessment terminology (Hamblin 2017; Sidi et al. 2019).

For staff who did not want students to access resources on the internet, a technological solution may have been the use of lockdown browsers. However, as noted by one lecturer, “*we weren’t allowed to use Respondus lockdown software, so students had the opportunity to search the internet for answers and ask each other*.” This staff member was associated with one of the 49% of Australian and New Zealand universities who chose not to deploy lockdown browsers to examinations conducted in the 2020 mid-year exams (Sankey 2020). In contrast, students commented on the ineffectiveness of lockdown browsers in an age where most students have access to multiple devices: “*People have multiple smart devices so lock down browsers do not help much at all and provide more problems than benefits*.” Concerns about student access to multiple devices are also identified in other studies (Brown 2018; Dendir and Maxwell 2020).

Students were also confused about whether the copy and paste function was permitted in online exams. For example, one student said: “*Well we were allowed notes, so it wasn’t technically cheating, but being able to do practice exams and then copy and paste the answers across made life easier*.” On the other hand, some lecturers thought that the “*ability to copy and paste from notes and website*” was cheating, regardless of the source. This point again highlights student and staff confusion about the specific practices that constitute breaches of academic integrity in their discipline and institution (Starovoytova and Arimi 2017).

#### Collusion

Most students and staff perceived that one of the main ways of cheating in online exams was through collusion as it was “*easy to share answers and communicate with other students*” through physical contact or using telecommunications. For example, one student said:

“*You could easily talk to peers over the phone or messaging during the exam, but on the other hand there was barely any time for such things. This would have only been effective if set up prior to the exam so that a small group of people can talk each other through the exam, but still write their own answers as answers were being compared*.”

Staff were also concerned that without monitoring “*there is no way to gauge/monitor the degree of collaboration during the task*” and compared this to the situation in a traditional invigilated exam. This concern about collusion is evident in the literature (Ray 2020; Sutherland-Smith 2013; Sutton and Taylor 2011), with a possible remedy being a shift to assessment that allow team work and collaboration (Bearman et al. 2020; Harper, Bretag, Rundle 2020; Harrison 2020; Ray 2020).

While the survey asked about students’ perceptions, not actual cheating behaviours, the survey yielded accounts from students of cheating: “I know people cheated because they spoke about it after the exam.”

Although it would be easy to collude with others during an exam, the value of doing so was questioned. As one student noted, while “*in most exams it was easy to ask for help from external sources, overall it did not affect answers given though as you still have to do them yourselves*.” Research indicates that collusion is common in Australian higher education (Sutherland-Smith 2013), with students from the business discipline more likely to engage in this form of academic misconduct that students from other disciplines (Sutton and Taylor 2011).

#### Impersonation

Some students and staff commented that technology could aid impersonation in online exams, as “*students can email, face time or send the whole exam to someone else to complete*.” A concern for academic staff was that “*there is no way to gauge/monitor if the work is the student’s own. While Turnitin is used to score similarity, in reality the student could have someone else write the whole document*.” This staff comment identifies one of the limitations of the use of text-matching software and raises the spectre of identify fraud as a problem in online exams (Lee-Post and Hapke 2017).

#### Contract cheating

The issue of contract cheating in online examination contexts was not commented on by students, but it was a concern raised by several lecturers. This may indicate that the use of contract cheating was not on the minds of students as they went into their first formal online examination. It may also reflect staff awareness of the prominent discourse around contract cheating in higher education (Bretag et al. 2019; Harper et al. 2020), which may have influenced their thinking about the online examination context. In the case of online assessments replacing invigilated exams some lecturers believed that “*contract cheating is easier*” as students have the “*opportunity to purchase a response*.” The increased possibility of contract cheating was perceived by staff to potentially occur when students have the time to engage in contract cheating by having “*questions in advance*.”

### Perceptions on what makes it easy to cheat in online exams

#### Lack of supervision

This study revealed that students and staff perceived lack of supervision to be a main factor in providing the opportunity for students to cheat, as has been found in other studies (Brown 2018; Cramp et al. 2019; Dendir and Maxwell 2020). Additionally, staff felt disempowered when decisions not to supervise online exams were “*made by the institution without any consultation with [the lecturer]*.” Indeed, one academic staff member stated that:

“*Non invigilation equates to license to cheat and collude. It signifies complete lack of control by the institution in maintaining the integrity of the exam. The aspect of timing the exam adds little to the confidence that the work submitted by the student is his/her own work. To some extent, it may make copying/colluding difficult but cannot eliminate it. At worst, the institution can never be sure that it is the student who has attempted the exam and not someone else, in some other part of the world. It makes a mockery of the whole exercise*.”

Similarly, a student commented that “*not having any kind of supervision makes it easy to cheat as the responsibility to keep their moral integrity is left completely to the students*.” Another student indicated “*It wasn’t supervised... I did not cheat but I am aware of many students who did*.”

The lack of supervision meant that students could potentially engage in a range of cheating behaviours, for example, “*[the exam] had components which were meant as closed book but not supervised which would have meant the dishonest people wanting to cheat very easily could have*.” A staff member echoed this comment by saying: “*Being uninvigilated there is the freedom to utilise smart phone and internet to communicate with others or to “google“ answers and to “copy/paste“ said answers into the exam text boxes.*”

Despite staff and student perceptions that lack of supervision made cheating easier, there were different perceptions of the efficacy of online proctoring. For example, one staff member observed that “*proctoring has been tried in the past and was found not to work as intended*,” and a student stated: “*I would say the camera doesn’t pick up everything all the time. I could see that it would’ve been easier for students to cheat if they wanted to*.”

Another staff member indicated that the failure of online proctoring was evident in *“results [that] were heavily skewed towards a better than normal result with higher grades being achieved.”* ​ This same lecturer commented that there was evidence of this assertion “*from an expert panel in an Accounting Education Special Interest Group.”*

### Deterrents to cheating

Students and staff perceived that deterrents to cheating behaviours are monitoring, student beliefs, question design, exam duration and deployment and marking practices.

#### Monitoring

Given that lack of supervision in online exams and alternate assessments was perceived as a main cause of cheating, increasing online proctoring was viewed as a solution to curbing opportunities for academic misconduct, with a student indicating: “*While I understand the university’s concerns regarding the possibility of an increase in cheating due to non-supervised online exams, this is easy enough to overcome by engaging the services of a proctoring site such as ProctorU. I would like to see the university explore this option*.” This view is supported by evidence that students are more likely to cheat where the risk of being caught is high, as occurs when supervision is in place (Brown 2018; Cramp et al. 2019; Dendir and Maxwell 2020).

#### Student beliefs

Regardless of ease of cheating, many students indicated that their own moral compass and beliefs ruled out cheating. This aligns with studies that highlight the importance of values and beliefs in curbing academic misconduct (Hsiao 2015; Rundle et al. 2019; Sullivan 2016), and aligned to this, the role played by moral reminders (Grym and Liljander 2016) in emphasising a culture of integrity.

As one student noted: “*If students wanted to cheat I am sure they could. The same goes for the exam hall.*” The academic integrity statement students were required to acknowledge at University A tapped into student beliefs and reminded them of university expectations. One student said “*… it [puts it] front of mind for students that they are signing up to being honest*.”

Even when students had a moral or ethical position against cheating themselves, many were concerned about the possibilities of others cheating if there was the opportunity to do so which *“was not fair for those trying to do the right thing going up against people that did cheat*.” The literature indicates that the perception that peers are cheating may influence students to cheat themselves, in order to level the playing field (Holden et al. 2020), however, fear of detection may curb this. For example, one student indicated that they were aware that cheating *“could have serious ramifications should they subsequently seek admission to practice law.”*

#### Question design

Question design was perceived as an effective means of countering cheating in exams. Despite responses indicating that staff had little awareness of the role of exam design in reducing cheating, open question responses revealed that staff considered that cheating is impacted by “*the style of questions offered*” and that it was easy for students to cheat if “*questions [are] too simple e.g., multiple choice*.” Another stated it was easy for students to cheat if “*essay type descriptive questions*” were used, whereas it was harder to cheat if “*interpretation and calculations questions*” were used. These comments refer to the cognitive demands of the exam questions impacting on the ease of cheating and the need to *“test students on understanding of concepts which cannot be easily downloaded from the internet*.” In some instances, students noticed that exam questions changed with the move to an online format, with one stating “*I feel like the questions were more about applying knowledge, so it was hard to cheat. So, if you didn’t actually fully understand a concept you would not know the answer anyway.”*

As noted by one student:

“*cheating was lowered also through the types of questions that exams contained. Our unit coordinators later spoke of how they had to be very careful in wording and types of questions that they used in the exam, as somewhere on the internet, chances are, someone has already asked that question. They were prepared for the possibility of people cheating, and I think this helped combat it*”.

In this case the lecturer had taken the time to “*becom[e] familiar with writing questions suitable for online format*.”

#### Exam duration

Exam duration was perceived to be a factor that enabled or minimised cheating behaviours, with “*excessive amounts of time*” making cheating easier relative to “*the volume of questions needing a response​ [and] the length of or multi-parts in a question needing a response*.” As a result, “*it would have been hard to cheat simply because of the volume of questions. Had you spent time looking for answers from your text book or online you would have struggled to finish on time*.”

If well calibrated, “*the timed questions were perfectly timed so you could not look things up in the text books and purely relied on prior learnt knowledge.*” The importance of timing was also found in another study conducted during the pandemic (Ng 2020) to be an important factor in developing an online exam environment that minimises cheating, even in the context of unsupervised open-book exams.

However, students indicated that the timing was not always spot on, with many complaints from students about the exam time period being too short, but also from others who indicated that they were given what seemed to be an excessive time for a timed-online exam/online assessments. This led to confusion about how students felt they should approach the exam, whether additional time above what they would have expected in an exam should be used to edit and fully reference the exam as they would an assignment.

#### Online test environment deployment settings

Deployment settings were used by academic staff to reduce cheating behaviours where the exam was set up in the online test environment in the institutional LMS. These were primarily related to randomisation of questions and the use of backtracking in the exam. As indicated by Sullivan (2016) the choices that academics make about online exam deployment settings “can ease or complicate cheating” (p 197).

Students did not comment about randomisation of questions, presumably because this would not have been obvious to them unless they were comparing their exam with another student’s. On the other hand, lecturers noted that the use of questions banks combined with the randomisation of questions and answers made it harder for students to cheat. This aligns with studies indicating that personalisation of exams is a good strategy to reduce cheating (Manoharan 2019; Sullivan 2016).

Reducing opportunities for collusion was also achieved by setting up questions to be viewed one-at-a -time rather than all at once. This practice was widely criticised by students for removing their ability to plan the order in which they would answer the questions or go back and check their answers: “*Not being able to go back and forth with the exam questions took away the mojo of answering. I like to answer all the ones I know straight away and then come back to the ones I find more difficult*.”

#### Text-matching software and marking practices

The use of text-matching software can offer an effective deterrence strategy for cheating behaviour (Sullivan 2016). When used by staff in this study to deter cheating behaviours it had the desired effect on some students: “*knowing that the questions would be run through a plagiarisation (sic) scanner upon submission deterred cheating*.”

However, the effectiveness of text matching software was questioned by some staff who made comments about its lack of usefulness, for example, “*Safeassign software didn’t pick up likeness between papers in a meaningful way.”* This perception is supported by research which found errors in reports generated by text-matching software which resulted in “artificially inflated similarity scores” (Eaton et al. 2020) that required human review. As observed by Sullivan (2016) “the persistence and prevalence of cheating indicate that technology tools are ultimately ineffective” (p 197), whereas the use of “technology-centric tools” (Sullivan 2016 p 198) in combination with “complementary social methods to mediate catalysts of cheating” (Sullivan 2016 p 198) is more effective.

Some students felt that the integrity of the examination process would be maintained by lecturers who *“got to know students’ background and their style of writing/communication*” and were able to pick this up in the marking. As one student said, “*at the end of the day someone’s marks throughout the semester will help guide whether someone has cheated or not*.” This points to faulty student perceptions about how staff identify, evidence and prosecute academic integrity. Further, large classes and high levels of casual staff reduce the ability of academics to monitor academic integrity at an individual level (Sullivan 2016).

## Discussion

From the findings we make seven observations, which are discussed in this section.

1. Proctored exams were used across a range of disciplines, even where this was not required by professional bodies.

Students from a wide range of disciplines at university C, the only participating university to use online proctoring, indicated that they had sat for online proctored exams. This was despite that university indicating that the use of online proctoring was limited to disciplines where it was required by an accrediting body. That exams were proctored in disciplines where there was no requirement to do so aligns with concerns expressed by staff about the integrity of online exams and the importance of supervision to deter cheating behaviours, despite evidence that contradicts this (Allan 2020; Cramp et al. 2019; Grajek 2020; James 2016).

2. The perception of students that cheating is harder in online examinations than in traditional invigilated exams contrasts with the perceptions of academic staff.

Students who sat for online examinations or alternative assessments considered that cheating would be harder in these online formats that in traditional invigilated exams. This aligns with literature about cheating in online environments (Sidi et al. 2019), but contrasts with the perceptions of academic staff who felt that students would find it easier to cheat in online examinations and alternative assessments than in traditional invigilated exams.

3. Students’ perceptions of the ease of cheating in online exam formats varied with age, while other demographic factors had no discernible impact.

Previous studies of student cheating behaviours have shown evidence of increased cheating aligned with an array of factors such as age, gender, discipline (Bretag et al. 2019). In this study, we found that age was the only factor that impacted on the perception of ease of cheating in online environments. This suggests that the characteristics of students who engage in cheating behaviours may differ between face-to-face and digital contexts.

4. Students and staff are confused about what constitutes cheating in online examinations.

This study found considerable confusion about what behaviours constitute cheating in digital exam and assessment environments and particularly about what resources could be accessed during an exam. This confusion was compounded by lack of clear instructions about what resources students could use during exams. This concern about accessing prohibited resources during an exam is not a recognised form of academic misconduct (INQAAHE 2020). Hence, cheating behaviours need to be redefined for the digital age (Evering and Moorman 2012), as do the terms used to described access to resources, such as ‘open’ and ‘closed’ book exams that are ambiguous in technology rich contexts.

The concept of ‘closed book’ is arguably redundant in the digital age, given that information is widely available and easy to access. Students ability to use information, rather than trying to restrict access to it, is what needs to be assessed. Given that it is not possible to stop students accessing online resources, it would make sense to design assessments that assume students do have access to a wide range of materials and resources.

5. Strong individual beliefs and values about integrity reduce the likelihood that students will cheat, as long as the examination and assessment environment is considered to be fair.

Academic integrity was important to staff and students, with students indicating strongly that they would not engage in cheating behaviours regardless of whether it was easy to cheat or not. This suggests that appealing to students’ values and beliefs with clear messaging about academic integrity could be an important strategy in enhancing the integrity of digital examinations and online assessments as well as promoting confidence in the integrity of the online exam environment.

6. Effective question design focused on high order thinking and designed for the digital environment is an essential requirement for maintaining academic integrity in digital assessment.

While the importance of question design and the use of high order thinking skills in assessment is well recognised (Sullivan 2016), this study pointed to additional considerations in digital exams and assessment. While testing low order cognition in face-to-face environments equates to memory testing, in digital environments students can quickly and easily search online for answers. Therefore, questions that require students to use information, rather than regurgitate it, are needed in the digital age.

7. The use of function resisting assessment practices such as online proctoring, lockdown browsers, and limiting backtracking in exams set in an online test environment do not necessarily have the intended outcome and may have unintended consequences.

There is evidence that technologies used to limit access to information can be thwarted by students who have the intention to cheat (Blumenfeldwitz 2020; Joel 2020). Each technology that restricts one aspect of student behaviour also has other consequences. One example from this study was the unintended consequences on students’ exam strategies and time management during exams resulting from the use of deployment settings not allowing backtracking in digital exams. The questionable effectiveness of tools such as online proctoring in controlling or securing online exam environments raises questions as to whether invigilation is redundant in the digital age. Rather than attempting the challenging and intrusive task of online proctoring to attempt to limit collusion and identity fraud in online exams, a better approach may be to design exams where cooperation is allowed, aligning exams more closely the workplace contexts where students ultimately will be required to demonstrate their knowledge. Given the importance of teamwork and communication as twenty-first century employability skills, it seems that the focus on individual exam responses is outdated in many instances.

For many students the move to online exams was viewed as “the correct format in the current world climate” whereas other students were resistant to the changes and expressed a preference to go back to traditional examinations, indicating that they would “prefer to check [their] knowledge the traditional way.” In the post-pandemic world, it is unlikely that examinations will revert to their pre-2020 form as the impact of the crisis response will have unfrozen and changed institutional approaches to assessment (Mishra et al. 2020). Many universities will use the pandemic as a springboard to greater reliance on digital exams and alternative forms of assessment, not least because of the financial savings that can be achieved by “cutting logistics costs (physical exam delivery and transporting of papers and staff to and from exam centres)” (Lacey 2010 paragrah 19). One challenge will be in the reconceptualisation of digital exams and assessment that are fit for purpose in a digital age. This requires thoughtful and innovative assessment design and deployment, aligned to a teaching and learning approach to academic integrity (Bertram Gallant 2017; Brimble 2015) and a move away from outdated approaches to academic integrity that are not effective when translated to online contexts.

### Lessons learned

Drawing on the data and on the discussion, we present the lessons learned from this study, noting that very little guidance currently exists for exam and assessment design that reduces cheating. This is likely due to “the vast array of assessment types and the choices involved in their design” (Munoz and Mackay 2019 p 1). The myriad ways in which online exams can be designed means that the specific lessons learned in this study may not be applicable to other contexts or to all online exam types. Despite this, the lessons learned will contribute to a “more comprehensive understanding of design choices and their relationship to cheating behaviours” (Munoz and Mackay 2019 p 2).

#### Lesson 1

Communicate clear expectations to students of ethical behaviour during exams. This study supports research by Grym and Liljander (2016) that providing moral reminders reduces students’ likelihood of cheating.

#### Lesson 2

Replace terms such as ‘open book’ and ‘closed book’ with relevant terms for the digital age, define these terms and use them consistently. A framework of new terms requires research, clear definitions and wide acceptance in order to be used consistently. The authors put forward the following suggestions as a starting point for discussion:

- ‘print and digital resources permitted (no internet)’;
- ‘print and digital resources and internet use permitted’; and
- ‘no print or digital resources or the internet permitted’.

#### Lesson 3

Communicate with clear instructions to students about what resources they are permitted to access during online exams, rather than use terms such as ‘open book’ that are designed for face-to-face examination contexts. This would reduce student stress about whether they are in breach of academic integrity guidelines.

#### Lesson 4

Advertise approaches being used to deter and detect cheating (such as text-matching software), and the penalties for breaching academic integrity standards. As digital examinations were new at the universities involved in this study there were no ‘current practices’ or set standards in relation to deterrence and detection of breaches of academic misconduct in the online exam context. Research indicates that making students aware of detection approaches is a deterrent to cheating (Eaton et al. 2020; Munoz and Mackay 2019).

For example, there were no guidelines for the use of detection solutions such as text-matching software or of technical deterrents to apply in the deployment of multiple-choice online exams. In addition, although there was evidence that students were referred to academic integrity policy, there was no clear and succinct indication of the scale of penalties that would be applied if students were found to engage in academic misconduct.

#### Lesson 5

Avoid use of online proctoring software and other function resisting software (such as removing the backtracking option in online exams) unless there is a clear need for their use. While these technical solutions are applied for the purpose of reducing the opportunity for students to engage in academic misconduct, their use needs to be considered in light of digital and other equity issues they raise as well as concerns around privacy.

#### Lesson 6

Provide enough time in the exam to sample the students’ acquired knowledge and skills but not too much time to do the exam given the volume of learning being tested. Time restrictions are an accepted approach to reducing the opportunity for cheating (Munoz and Mackay 2019).

#### Lesson 7

Individualise exams set in the LMS test tool by using multiple question pools, randomising questions and answers, and/or to generate individualised questions using calculated formula settings, if relevant. As digital exams were new to staff implementing them at the universities involved in this study there were no ‘current practices’ to guide design or deployment approaches to individualise exams and maximise student opportunities to demonstrate knowledge while minimising the risk of breaches of academic integrity.

#### Lesson 8

Use hurdle tasks that require student to acknowledge academic integrity policies and the consequences of academic misconduct if caught prior to providing them with access to the online exam. As digital exams were new to staff at the universities involved in this study the use of hurdle tasks was not common across or within the universities. The use of hurdle tasks emphasising the requirement for academic integrity reinforces a culture of integrity.

#### Lesson 9

Design assessment tasks/questions that:

- Require students to utilise high level thinking skills, as these are less likely to be searchable online, and
- Allow students to utilise resources usually available in professional practice, such as typing rather than handwriting exams, and access to digital resources and the internet.

### Further research

This study points to areas of further research to deepen understanding and approaches to embedding academic integrity into the design, deployment and environment of online examinations.

- What, if any, are the impacts of different approaches to online assessment on the evaluation of learning outcomes?
- What, if any, are the disciplinary differences in student perceptions of cheating behaviours in online exams?
- What were the disciplinary differences in the take-up of proctoring by discipline, and what are the reasons for this?
- What tensions exist between systems-bureaucratic focus and academic approaches to the design and deployment of online assessment e.g. cost of delivery rather than quality of assessment?
- Why is handwriting still largely used in examinations in the digital age?
- How can academic integrity be better supported in the context of online examinations?

## Conclusion

Digital technologies including learning management systems are an integral part of universities’ virtual learning environments, so it makes sense that the enforced experiment of online examinations during the pandemic will leave a legacy post-pandemic. Whether universities return wholesale to face-to-face examinations is unlikely but remains to be seen. This study into the experiences and perceptions of academic staff and students shows that what constitutes academic integrity needs to be reassessed for a digital world.

We have observed that cheating occurs in all forms of online examinations whether proctored or non-proctored, just as it does in face-to-face examinations. The current debate tends to ignore this fact and focuses on transferring approaches designed for minimising cheating in face-to-face environments into the very different context of digital exams and assessment. This study supports an integrated approach to minimising cheating in online environments (Sullivan 2016) that combines a focus on assessment design (Brimble 2015) with strengthening a culture of integrity and utilising the affordances of technology. This combination of approaches is likely to be more effective to reducing academic misconduct than technical solutions alone (Sullivan 2016). The seven main observations drawn from the study and the lessons learned from it provide practical strategies for creating a culture of academic integrity around digital assessment as we move towards normalising online examinations and assessment post-pandemic. In addition, there is a need for continuing research of how academic integrity in online examinations can be better achieved.

## Data Availability

The resources described in this paper are not publicly available.

## Appendix 1

**Table 2:** Student Survey Questions and Branch Conditions

Question Number	Question Type (inc. conditional branch origin)	Question	Response Input Field or Selection
Q1.	Default	Please select your age range.	• Under 18 • 18–24 • 25–34 • 35–44 • 45–54 • 55–64 • 65–74 • 75–84 • 85 or older
Branch Condition		If “*Under 18*” selected, branch to end of survey.	
Q2.	Default	I study at:	• University 1^a^ • University 2^a^ • University 3^a^
Q3.	Default	My academic discipline is (e.g. Management, Finance, Engineering, Psychology...):	Open response [Multi-line editable text field supplied]
Q4.	Default	My level of study is:	• Undergraduate • Postgraduate
Q5.	Default	I am a:	• Domestic Student • International Student
Q6.	Default	My gender:	• Male • Female • Non-binary
Q7.	Default	My usual mode of study prior to COVID-19 was:	• Internal (campus based) • External (online)
Q8.	Default	Thinking about one of the standard invigilated exams (e.g. in an exam centre) planned (prior to COVID19) for this teaching period, now select the assessment format that replaced it:	• Alternate written assessment (e.g. like a mid-semester essay/assignment) • Timed online/take-home exam (non-invigilated) • Timed online exam invigilated (proctored, e.g. with camera) • Other
Branch Condition		If “*Timed online/take-home exam (non-invigilated)*” selected, allow Q9.	
		If “*Timed online/take-home exam (non-invigilated)*” selected, allow Q15.	
		If “*Timed online exam invigilated (proctored,* e.g. *with camera)*” selected, allow Q11.	
		If “*Timed online exam invigilated (proctored,* e.g. *with camera)*” selected, allow Q13.	
		If “*Timed online exam invigilated (proctored,* e.g. *with camera)*” selected, allow Q15.	
		If “*Other*” selected branch, to Q14.	
Q9.	Conditional - Q8.	Were you given access to a sample ‘Timed online/take-home exam (non-invigilated)’ to practice downloading, completing and submitting before the exam period?	• Yes • No
Branch Condition		If “*Yes*” selected, allow Q10.	
Q10.	Conditional - Q9.	Did you take time to complete the practice ‘Timed online/take-home exam (non-invigilated)’ process?	• Yes • No
Q11.	Conditional - Q8.	Were you given access to a sample ‘Timed online exam invigilated (proctored, e.g. with camera)’ to practice downloading, completing and submitting before the exam period?	• Yes • No
Branch Condition		If “*Yes*” selected, allow Q12.	
Q12.	Conditional - Q11.	Did you take time to complete the practice ‘Timed online exam invigilated (proctored, e.g. with camera)’ process?	• Yes • No
Q13.	Conditional - Q8.	Please describe any privacy or other concerns you had with the online invigilation/proctoring process or technologies.	Open response [Multi-line editable text field supplied]
Q14.	Conditional - Q8.	Please describe the format of other assessment used.	Open response [Multi-line editable text field supplied]
Q15.	Conditional - Q8.	Did you have any issues with your internet connection during the online exam?	• Yes • No
Branch Condition		If “*Yes*” selected, allow Q16.	
Q16.	Conditional - Q15.	Please describe the internet issues you had during the online exam.	Open response [Multi-line editable text field supplied]
Q17.	Default	Select the type of device you used to complete the online exam:	• Desktop PC • Laptop • Tablet • Smart Phone • Other
Branch Condition		If “*Other*” selected branch, to Q18.	
Q18.	Conditional - Q17.	Please describe the device you used to complete the online exam.	Open response [Multi-line editable text field supplied]
Q19.	Default	Did you have any issues with technology (e.g. Computer, Laptop, Internet …) during the online exam?	• Yes • No
Branch Condition		If “*Yes*” selected, allow Q20.	
Q20.	Conditional - Q20.	Please describe the technology (e.g. Computer, Laptop, Internet …) issues you had during the online exam?	Open response [Multi-line editable text field supplied]
Q21.	Default	Compared to a standard invigilated exam (e.g. in an exam centre) the alternate assessment or online exam was more stressful.	• Strongly agree • Agree • Somewhat agree • Neither agree nor disagree • Somewhat disagree • Disagree • Strongly disagree
Q22.	Conditional - Q21.	Please describe the factors that either increased or decreased your stress.	Open response [Multi-line editable text field supplied]
Q23.	Default	The online exam or alternate assessment made cheating ______________________:	• Very easy • Fairly easy • Somewhat easy • Neither easy nor hard • Somewhat hard • Fairly hard • Very hard
Branch Condition		If “*Neither easy nor hard*” is not selected, allow Q24.	
Q24.	Conditional - Q23.	What factors do you think make it easy or hard to cheat in the online exam or alternate assessment.	Open response [Multi-line editable text field supplied]
Q25.	Default	Select the exam or alternate assessment format you would prefer in the future:	• Traditional exam in an exam centre. • Alternate written assessment (e.g. like a mid-semester essay/assignment). • Online timed exam non-invigilated. • Online timed exam invigilated (proctored).
Q26.	Default	Did you have any issues with the format (e.g. Word or PDF document, online test tool …), of the online exam or alternate assessment:	• Yes • No
Branch Condition		If “*Yes*” selected, allow Q27.	
Q27.	Conditional - Q26.	Please describe the issues you experienced with the format of the online exam.	Open response [Multi-line editable text field supplied]
Q28.	Default	The transition to the alternate assessment or online exam format was communicated clearly and consistently.	• Strongly agree • Agree • Somewhat agree • Neither agree nor disagree • Somewhat disagree • Disagree • Strongly disagree
Q29.	Default	Please outline the Positive aspects of the online exam or alternate assessment format you experienced.	Open response [Multi-line editable text field supplied]
Q30.	Default	Please outline the Negative aspects of the online exam or alternate assessment format you experienced.	Open response [Multi-line editable text field supplied]
Q31.	Default	Please provide any other comments or suggestions you have about the online exam or alternate assessment you experienced.	Open response [Multi-line editable text field supplied]

^a^Names of the universities have been removed for publication

## Appendix 2

**Table 3:** Staff Survey Questions and Branch Conditions

Question Number	Question Type (inc. conditional branch origin)	Question	Response Input Field or Selection
Q1.	Default	Due to COVID19 did you have to move from a standard invigilated exam (e.g. in an exam center) to an alternate assessment format?	• Yes • No
Branch Condition		If “*No*” selected, branch to end of survey.	
Q2.	Default	I teach at:	• University 1^a^ • University 2^a^ • University 3^a^
Q3.	Default	My academic discipline is (e.g. Management, Finance, Engineering, Psychology...):	Open response [Multi-line editable text field supplied]
Q4.	Default	My gender:	• Male • Female • Non-binary
Q5.	Default	I normally teach in this mode (multiple selections allowed):	• Internal campus based • External online
Q6.	Default	I am confident in teaching and assessment using online technology	• Strongly agree • Agree • Somewhat agree • Neither agree nor disagree • Somewhat disagree • Disagree • Strongly disagree
Q7.	Default	Select one assessment format type that replaced your standard invigilated exam (e.g. in an exam centre):	• Alternate written assessment (e.g. like a mid-semester essay/assignment) • Timed online/take-home exam (non-invigilated) • Timed online exam invigilated (proctored) • Other
Branch Condition		If “*Other*” selected branch, to Q8.	
Branch Condition		If “*Alternate written assessment (*e.g. *like a mid-semester essay/assignment)*” selected branch, to Q9.	
Branch Condition		If “*Alternate written assessment (*e.g. *like a mid-semester essay/assignment)*” selected branch, to Q10.	
Branch Condition		If “*Timed online/take-home exam (non-invigilated)*” selected branch, to Q11.	
Branch Condition		If “*Timed online/take-home exam (non-invigilated)*” selected branch, to Q12.	
Branch Condition		If “*Timed online exam invigilated (proctored)*” selected branch, to Q13.	
Branch Condition		If “*Timed online exam invigilated (proctored)*” selected branch, to Q14.	
Branch Condition		If “*Other*” selected branch, to Q15.	
Branch Condition		If “*Other*” selected branch, to Q16.	
Q8.	Conditional - Q7.	Briefly describe the ‘Other’ assessment format you replaced the standard invigilated exam with.	Open response [Multi-line editable text field supplied]
Q9.	Conditional - Q7.	Student cheating is minimised by the format of the “Alternate written assessment (e.g. like a mid-semester essay/assignment)”.	• Strongly agree • Agree • Somewhat agree • Neither agree nor disagree • Somewhat disagree • Disagree • Strongly disagree
Q10.	Conditional - Q7.	Please describe the factors that you think make it easier or harder to cheat in the “Alternate written assessment (e.g. like a mid-semester essay/assignment)” format.	Open response [Multi-line editable text field supplied]
Q11.	Conditional - Q7.	Student cheating is minimised by the format of the “Online timed exam non-invigilated” format.	• Strongly agree • Agree • Somewhat agree • Neither agree nor disagree • Somewhat disagree • Disagree • Strongly disagree
Q12.	Conditional - Q7.	Please describe the factors that you think make it easier or harder to cheat in the “Online timed exam non-invigilated” format.	Open response [Multi-line editable text field supplied]
Q13.	Conditional - Q7.	Student cheating is minimised by the format of the “Online timed exam invigilated (proctored)”.	• Strongly agree • Agree • Somewhat agree • Neither agree nor disagree • Somewhat disagree • Disagree • Strongly disagree
Q14.	Conditional - Q7.	Please describe the factors that you think make it easier or harder to cheat in the “Online timed exam invigilated (proctored)” format.	Open response [Multi-line editable text field supplied]
Q15.	Conditional - Q7.	Student cheating is minimised by the format of the “Other” assessment.	• Strongly agree • Agree • Somewhat agree • Neither agree nor disagree • Somewhat disagree • Disagree • Strongly disagree
Q16.	Conditional – Q7.	Please describe the factors that you think make it easier or harder to cheat in the “Other” assessment format.	Open response [Multi-line editable text field supplied]
Q17.	Default	I was given clear guidance on what alternate assessment formats could be used?	• Strongly agree • Agree • Somewhat agree • Neither agree nor disagree • Somewhat disagree • Disagree • Strongly disagree
Q18.	Default	If due to COVID19 you can’t use standard invigilated exams (e.g. in an exam centre) in Semester 22,020, what is your preferred final assessment format:	• Alternate written assessment (e.g. like a mid-semester essay/assignment). • Online timed exam non-invigilated. • Online timed exam invigilated (proctored). • Other
Branch Condition		If “*Other*” selected, allow Q19.	
Branch Condition		If “*Alternate written assessment (*e.g. *like a mid-semester essay/assignment)*” selected, allow Q20.	
Branch Condition		If “*Alternate written assessment (*e.g. *like a mid-semester essay/assignment)*” selected, allow Q23.	
Branch Condition		If “*Online timed exam non-invigilated*” selected, allow Q21.	
Branch Condition		If “*Online timed exam non-invigilated*” selected, allow Q24.	
Branch Condition		If “*Online timed exam invigilated (proctored)*” selected, allow Q22.	
Branch Condition		If “*Online timed exam invigilated (proctored)*” selected, allow Q25.	
Q19.	Conditional - Q18.	Briefly describe the ‘Other’ assessment format you would replace the standard invigilated exams (e.g. in an exam centre).?	Open response [Multi-line editable text field supplied]
Q20.	Conditional - Q18.	Please briefly describe the things you did as a teacher to help prepare students for the “Alternate written assessment (e.g. like a mid-semester essay/assignment)” assessment.	Open response [Multi-line editable text field supplied]
Q21.	Conditional - Q18.	Please briefly describe the things you did as a teacher to help prepare students for the “Online timed exam non-invigilated” assessment.	Open response [Multi-line editable text field supplied]
Q22.	Conditional - Q18.	Please briefly describe the things you did as a teacher to help prepare students for the “Online timed exam invigilated (proctored)” assessment.	Open response [Multi-line editable text field supplied]
Q23.	Conditional - Q18.	Please briefly describe what your university did to help prepare students for the “Alternate written assessment (e.g. like a mid-semester essay/assignment)” assessment.	Open response [Multi-line editable text field supplied]
Q24.	Conditional - Q18.	Please briefly describe what your university did to help prepare students for the “Online timed exam non-invigilated” assessment.	Open response [Multi-line editable text field supplied]
Q25.	Conditional - Q18.	Please briefly describe what your university did to help prepare students for the “Online timed exam invigilated (proctored)” assessment.	Open response [Multi-line editable text field supplied]
Q26.	Default	Moving to the different assessment format resulted in a ___________________ in my workload:	• Significant increase • Moderate increase • Slight increase • Neither increase nor decrease • Slight decrease • Moderate decrease • Significant decrease
		If “*Neither increase nor decrease*” selected, allow Q27.	
Q27.	Conditional - Q26.	Briefly list the reasons for this change in your workload	Open response [Multi-line editable text field supplied]
Q28.	Default	Did you need to learn any new skills or update old skills in order to develop and deploy the online exam or alternate assessment?	• Yes • No
		If “*Yes*” selected, allow Q29.	
Q29.	Conditional - Q28.	Briefly list the skills you needed to learn of update.	Open response [Multi-line editable text field supplied]
Q30.	Default	I was given sufficient support to develop and deploy the online exam or alternate assessment?	• Strongly agree • Agree • Somewhat agree • Neither agree nor disagree • Somewhat disagree • Disagree • Strongly disagree
		If “*Somewhat disagree” or “Disagree” or “Strongly disagree*” selected, allow Q31.	
Q31.	Default	Please briefly describe the support you think you should have been given.	Open response [Multi-line editable text field supplied]

^a^Names of the universities have been removed for publication

## Abbreviations

INQAAHE: International Network for Quality Assurance Agencies in Higher Education
LMS: Learning management system
TEQSA: Tertiary Education Quality and Standards Organisation
